# Investigation of SOSTDC1 gene in non-syndromic patients with supernumerary teeth

**DOI:** 10.4317/medoral.22520

**Published:** 2018-09-28

**Authors:** Volkan Arikan, Ozge Cumaogullari, Betul-Memis Ozgul, Firdevs-Tulga Oz

**Affiliations:** 1 DDS, PhD, Assistant Professor, Department of Pediatric Dentistry, Faculty of Dentistry, Kirikkale University, Kirikkale, Turkey; 2 PhD, Biotechnology Institute, Ankara University, Ankara, Turkey; 3 DDS, PhD, Assistant Professor, Department of Pediatric Dentistry, Faculty of Dentistry, Baskent University, Ankara, Turkey; 4 DDS, PhD, Professor, Department of Pediatric Dentistry, Faculty of Dentistry, Ankara University, Ankara, Turkey

## Abstract

**Background:**

The etiology of supernumerary teeth is still unclear however heredity is believed to be a major factor and this idea was supported by several case reports. Recently, a relationship between supernumerary tooth formation and deficiency of Uterine Sensitization Associated Gene-1 (Usag-1), a rat gene that is expressed in sensitized endometrium, was reported in mice. The human homolog gene for Usag-1, Sclerostin Domain Containing 1 (SOSTDC1), shows 85% identity with mouse Usag-1. The present study aimed to investigate SOSTDC1 coding regions in non-syndromic patients with one or more supernumerary teeth.

**Material and Methods:**

Twenty-five non-syndromic patients (21 male and 4 female) aged 5-15 years, with one or more supernumerary teeth were included in the study. Saliva samples were collected from patients and DNA samples were isolated and analyzed using PCR.

**Results:**

Eight phenotypes of supernumerary tooth formation were observed in the study. From the DNA analysis, 2 novel and 3 previously identified sequence alterations were identified however, in investigating the Usag-1 homolog SOSTDC1 gene, the present study could not find any phenotype-genotype relationship.

**Conclusions:**

There are many SOSTDC1 homolog genes in the human genome and future studies should investigate these candidate genes. Also studies in larger case groups including family members may reveal the hereditary pattern.

** Key words:**Genetics, Usag-1, mesiodens, DNA sequencing, pediatric dentistry, PCR.

## Introduction

Teeth produced in greater numbers than the normal dental formula are referred to as supernumerary teeth (1). The etiology of supernumerary teeth is still unclear, although there are some theories regarding the mechanism of their formation, including genetic and environmental factors (2). While this anomaly is most commonly observed in the premaxilla, it can be found everywhere in the dental arch, and can occur in both primary and permanent dentition (3,4). Its prevalence ranges from 0.5% to 3.8% in permanent dentition and from 0.3% to 1.9% in primary dentition (5-8).

Heredity is believed to be a major factor behind supernumerary tooth formation. It has been suggested that supernumerary teeth may be associated with autosomal recessive heredity, with lower penetrance in females (9). However, a few case reports have also proposed a low frequency autosomal dominant inheritance of this phenotype (10-12). Several articles support the idea that genetic component is needed for development of supernumerary teeth (13-15).

Recently, a relationship between supernumerary tooth formation and deficiency of Uterine Sensitization Associated Gene-1 (*Usag-1*), a rat gene that is expressed in sensitized endometrium (16), was reported in mice (17-19). Previous studies have shown that *Usag-1* and its human orthologous (ectodin) binds, neutralizes and acts as an antagonist of morphogenetic proteins (BMP). It has also been reported to inhibit Wnt signaling (18,20,21) while it is well established that both BMP and Wnt play a role in tooth morphogenesis (22,23), making it is very likely that *Usag-1* has a role in supernumerary teeth formation.

The human homolog gene for *Usag-1*, Sclerostin Domain Containing 1 (*SOSTDC1*), shows 85% identity with mouse *Usag-1*. The gene is localized on 7p21.1 with two transcripts of 3 and 5 exons, coding protein products of 206 and 230 amino acids respectively.

This study is the first investigation of *SOSTDC1* in humans with supernumerary teeth phenotype. We aimed to investigate *SOSTDC1* coding regions in non-syndromic patients with one or more supernumerary teeth.

## Material and Methods

Twenty-five non-syndromic patients who were recruited through Ankara University’s Department of Pediatric Dentistry between January 2011 and January 2013 with one or more supernumerary teeth were included in the study. There were 21 male and 4 female patients, aged 5-15 years. Additional patient information is given in the  Table 1. Ethical approval was received from the Institutional Review Board (144/4), and informed consent was obtained from all participants and their parents.

**Table 1:**
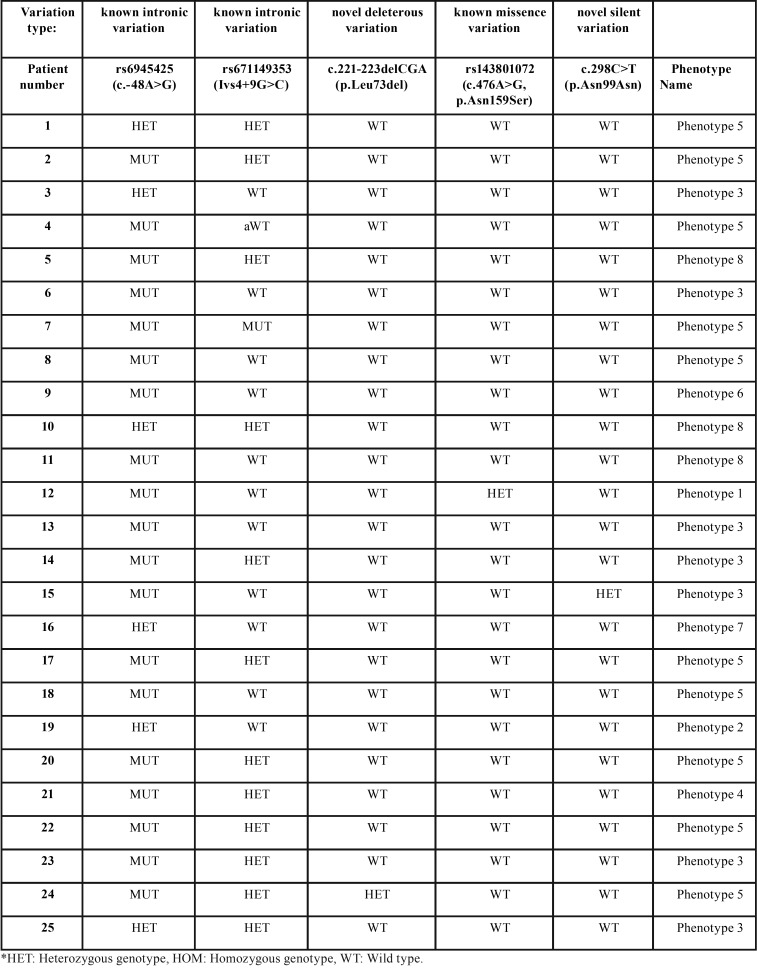
DNA analysis and additional patient information.

- DNA isolation

Samples were collected from patients’ saliva. Before isolating saliva DNA, the samples were dissolved in 10 ml isotonic solution in 15 ml falcon tubes. The tubes were centrifuged at 400g for 10 minute. Afterwards, the pellets were incubated at 56°C for 10 min, in 180 μl dH2O and 20 μl proteinase K (20mg/ml). DNA isolations were performed using QIAamp DNA Blood Mini Kit (Qiagen Inc.) kit according to the manufacturer’s instructions. DNA samples were spectrophotometrically analyzed and stored at -200C.

- Polymerase Chain Reaction (PCR)

To amplify the SOSTC1 gene (ENST00000396652), 3 pairs of primers were designed for coding exons ( Table 2). Optimum PCR condition was obtained with 10 pmol primer mix, 0.5 unit SuperHot Taq polymerase enzyme (Bioron Inc.), and 1X Buffer Complete (Bioron Inc.) in 25 μl total volume. For each PCR reaction, a 20-50 ng/µl DNA template was used. Primer annealing temperature was optimized to 60°C. Thermal cycling conditions were 95°C 10 min for 1 cycle, 95°C 45 sec, 60°C 45 sec, 72°C 45 sec for 35 cycles, and 72°C 10 min. The PCR product was loaded in 2% agarose gel. Amplicon sizes of the PCRs were 531 bp (exon 3), 354 bp (exon 4) and 597 bp (exon 5).

**Table 2:**
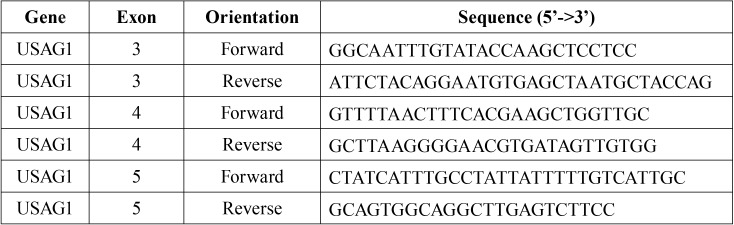
Sequences of primers used of the amplification of SOSTC1 gene.

- PCR Purification and DNA Sequencing

All PCR products were purified by using NucleoFast® 96 PCR kit (Macherey-Nagel GmbH). DNA sequencing was performed by cycle sequencing in 20 μl total volume. Sequencing reactions were set with both forward and reverse primers. Purification of the sequencing reaction was performed with ZR-96 DNA Sequencing Clean-up Kit (Zymo Research Corp.) according to the manufacturer’s recommendations. Capillary electrophoresis was performed by ABI 3130 capillary electrophoresis instrument (Applied Biosystems Inc.). Electrophoregrams were analyzed by using SeqScape 2.5.0 software (Applied Biosystems Inc.).

- Bioinformatics analysis

DNA sequence results of the patients were aligned to Ensembl Grch37 Homo sapience SOSTDC1 coding regions nucleotide sequences to determine alterations. Subsequently, alterations were compared to “NCBI/NIH dbSNP (The Short Genetic Variations Database) short variations catalogs Homo sapience dataset”(24). The effect of missense mutation alterations in protein were investigated with SIFT analysis (25,26).

## Results

A total of 25 patients (21 male, 4 female) were examined, of which 9 had single supernumerary teeth while the rest had 2 or more. Among the patients with single supernumerary teeth, 7 had mesiodentes ( Table 3). Eight phenotypes were observed in our cohort ( Table 4).

**Table 3:**
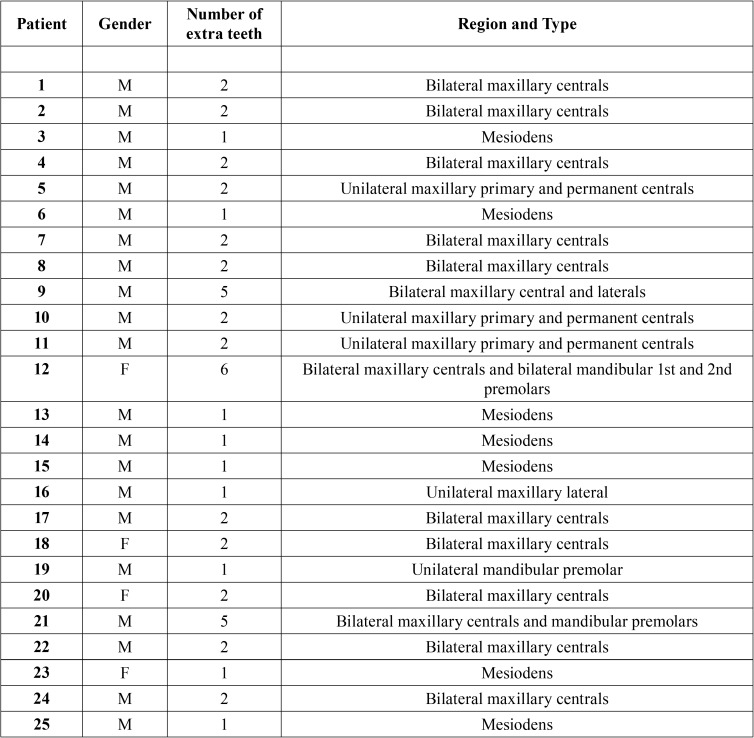
Distribution on number, region and types of supernumerary teeth.

**Table 4:**
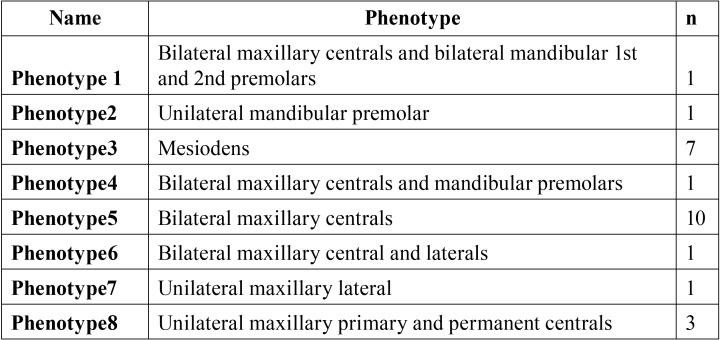
Phenotypic variations.

From the DNA analysis, we identified 2 novel and 3 previously identified sequence alterations ( Table 5). The three previously discovered SNPs (Single Nucleotide Polymorphisms) are rs6945425, rs67149353, rs143801072. For the first two of these, the Minor Allele Frequencies (MAF) were 0.1212 and 0.2084 respectively in the dbSNP database. rs6945425 (c.-48A>G) and rs67149353 (IVS4+9G>C) are intronic nucleotide substitutions with no functional consequences on the protein. According to dbSNP, these SNPs have not been related with any syndrome. On the other hand, rs143801072 (c.476A>G, N159S), which shows a very low frequency in other Caucasian populations (0.010), is a missense mutation causing a change from asparagine to serine. We found rs143801072 in only one patient with phenotype 1 in heterozygous state, showing that the Asp>Ser amino acid alteration was tolerated by the protein. This patient was also found to carry rs6945425 variation in homozygous state.

**Table 5:**
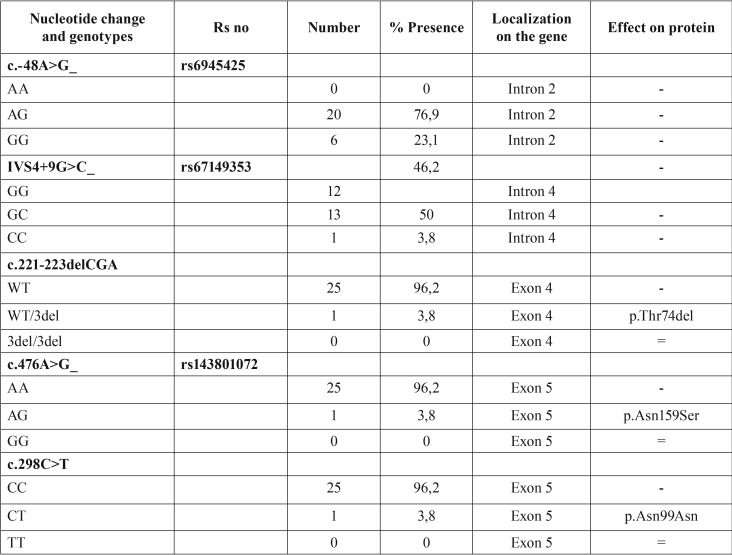
Identified alterations in our study.

The 2 novel mutations we identified had heterozygous genotype in phenotypes 3 and 5. One of these alterations, c.221-223delCGA, was an in-frame deletion causing one tyrosine amino acid to be deleted in SOSTDC1 ( Table 1). This was detected in 1 in 10 patients with phenotype 5.

The other novel nucleotide alteration, c.298C>T, is a synonymous mutation that does not change asparagine amino acid at residue 99. This substitution was identified in 1 in 7 cases with phenotype 3.

## Discussion

The etiology behind the formation of supernumerary teeth still remains unknown. However, several theories have been investigated previously. One of these theories was dichotomy, which claims that the developing tooth bud may be divided to form a supernumerary tooth (27). Hyperactivity of the dental lamina has also been suggested as a possible factor behind the formation of supernumerary teeth (4).

Various genes (*RUNX2, PLOD, EVC, GLA, APC, NEMO*) have been associated with supernumerary teeth formation in several syndromes, such as Cleidocranial dysplasia, Ehlers-Danlos Type IV, Ellis-Van Creveld, Fabry disease, Familial adenomatous polyposis, and Incontinentia pigmenti (13,28,29). As previously noted, our study focused on non-syndromic supernumerary tooth formation.

In the last two decades, several studies conducted on mutation induced mice in *Usag-1, Gas1, Eda, Spry 2, Spry 4,* and *Pax 6* resulted in supernumerary tooth formation (14,15,17,30-33).

Even though the etiology behind supernumerary teeth cannot be clearly determined, studies have shown that cell cycle related pathways like WNT, MAPK/ERK, and PI3K/AKT/Mtor are involved in supernumerary tooth formation. It is well known that BMP and Wnt are key molecules controlling tooth morphogenesis (22). BMP is known to regulate embryonic development in all animals, being present in practically all tissues and organs. *Usag-1* (also known as *Ectodin, Sostdc1* or *Wise*), which is expressed in the epithelium and mesenchyme of the developing tooth germ, encodes a secreted BMP-inhibitor (21,34). Recently, Murashima-Suginami *et al.* have shown that *Usag-1* abrogation in mice resulted in the survival of rudiment incisors and formation of supernumerary teeth (18). In a different study, their team also found that BMP signaling increases in *Usag-1* deficient mice since this gene is an antagonist of BMPs, which results in the formation of supernumerary teeth (19). According to Kiso *et al.*, as a result of *Usag-1* gene deficiency, due to lack of apoptotic elimination, odontogenic mesenchymal cells were retained in mice, while the interaction between *Bmp-7* and *Usag-1* had a role in the formation of supernumerary organs (35). Kassai *et al.* also reported supernumerary tooth formation in *Usag-1* (which they named Ectodin) deficient mice (17).

In this study, we investigated *Usag-1* gene homolog SOSTDC1 in 25 patients with at least one supernumerary tooth without any syndromes. The majority (84%) of our study group were male patients. We detected 2 novel and 3 previously identified nucleotide alterations ( Table 4).

The known variations rs6945425 (c.-48A>G) and rs67149353 (IVS4+9G>C) are intronic nucleotide substitutions with no functional consequences on the protein whereas, as previously noted, rs143801072 (c.476A>G) is a missense mutation (p.Asn159Ser). This residue (p.Asn159Ser) is found to be conserved in many species ( Table 6). MAF is 0.01 among Caucasian populations. This residue lies within the evolutionarily conserved C-terminal cystine knot-like (CTCK) domain. As in other hereditary diseases, such as Bardet Biedl and Meckel Syndrome, hereditary non-syndromic supernumerary phenotype seems to be multigenic. Thus, this rare SNP in a functional domain in a very highly conserved residue may cause supernumerary teeth formation in just 1 in 10 patients with phenotype 1. It is important to emphasize that phenotype 1 was found in only one patient with rs143801072 heterozygous variation. However, this patient, for whom we lacked the family history, was the only case where we identified a potentially deleterious heterozygous mutation. It is therefore not possible to fully establish the relationship between supernumerary tooth formation, phenotype 1 and rs143801072. Besides, rs6945425 homozygous genotype was also detected in this patient, along with other 20 patients. Further investigations are therefore needed to elucidate the functional consequences of this missense mutation.

**Table 6:**
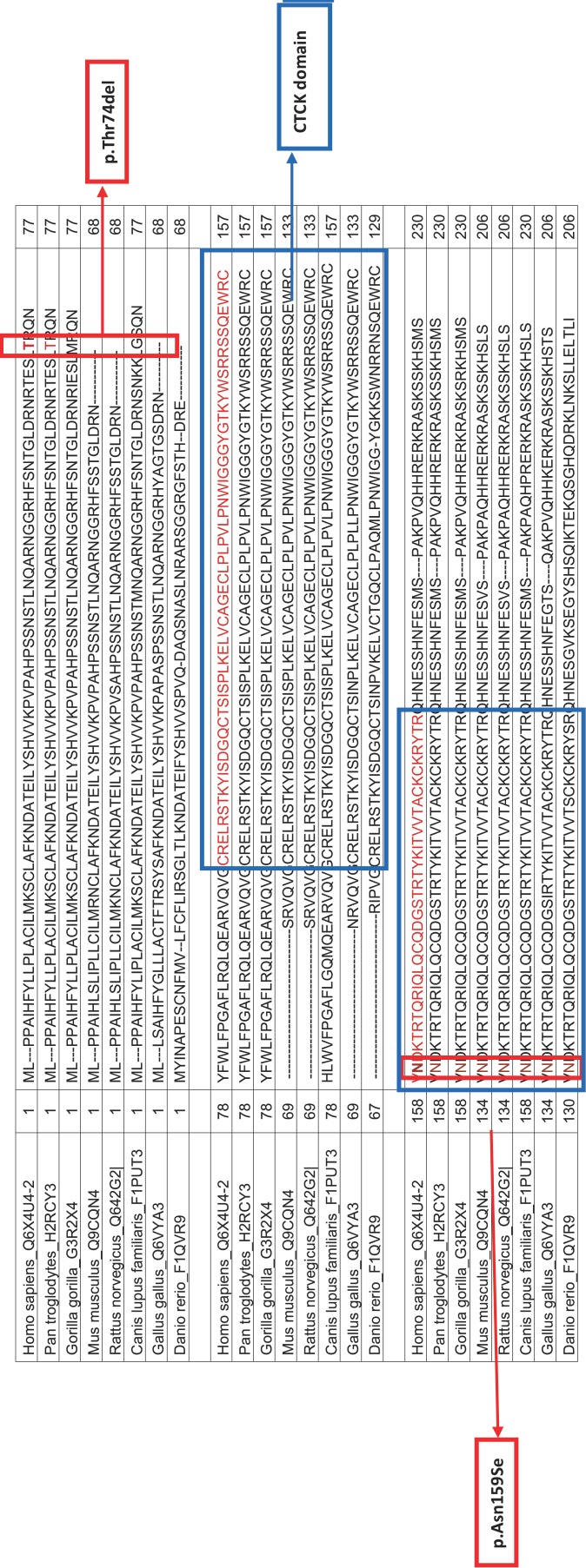
Comparison of SOSTDC1 protein in different species with protein domains and variation points.

We also identified 2 novel mutations, c.221-223delCGA and c.298C>T. One patient was heterozygous for the c.221-223delCGA alteration, which causes an in-frame deletion of tyrosine at residue 74 in the *SOSTDC1* protein ( Table 1). Further functional assays need to be conducted in order to establish whether this deletion impairs protein function. We evaluated probable functional consequences of this amino acid deletion in silico using three dimensional structure analysis tools (Coils regions, Domain linker prediction, Helical context). The analyses showed that the deletion of tyrosine at residue 74 did not have any structural or functional effect on the protein. Besides, this residue is not conserved across species. According to the sift analysis, this variation can be tolerated without affecting protein function.

The other novel nucleotide variation, c.298C>T, is a silent mutation that does not change asparagine at residue 99. Thus, this substitution has no functional impact on *SOSTDC1*.

In investigating the *Usag-1* homolog *SOSTDC1* gene, we could not find any phenotype-genotype relationship. According to our in silico analysis, there are many *SOSTDC1* homolog genes in the human genome ( Table 7). Future studies should investigate these candidate genes within the same study group. It would also be useful to enlarge the case group, and include family members, which may reveal the hereditary pattern.

**Table 7:**
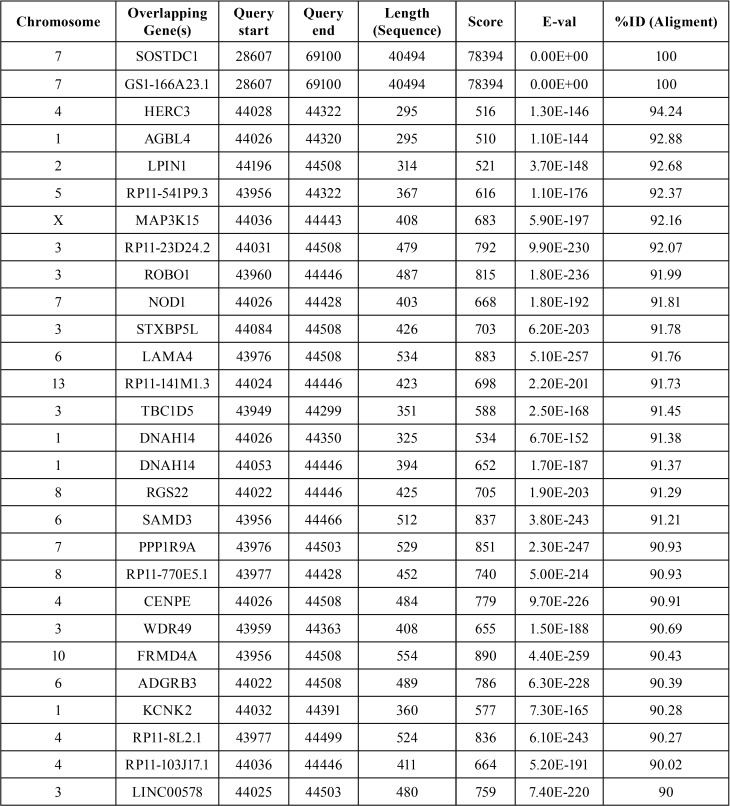
SOSTDC1 90% and more homologue genes in the human genome (Blast-Ensembl tool).
